# Mechanical Behaviour and Morphology of Thixoformed Aluminium Alloy Reinforced by Graphene

**DOI:** 10.3390/ma15196791

**Published:** 2022-09-30

**Authors:** Afifah Md Ali, Mohd Zaidi Omar, Mohd Shukor Salleh, Hanizam Hashim, Intan Fadhlina Mohamed, Nur Farah Bazilah Wakhi Anuar

**Affiliations:** 1 Department of Mechanical and Manufacturing Engineering, Faculty of Engineering and Built Environment, Universiti Kebangsaan Malaysia, Bangi 43600, Malaysia; 2 Department of Manufacturing Process, Faculty of Manufacturing Engineering, Universiti Teknikal Malaysia, Melaka 76100, Malaysia; 3 Department of Manufacturing Technology, Faculty of Mechanical and Manufacturing Engineering Technology, Universiti Teknikal Malaysia, Melaka 76100, Malaysia

**Keywords:** aluminium, thixoforming, microstructure, graphene nanoplatelets

## Abstract

Thixoforming is a promising method that offers several advantages over both liquid and solid processing. This process utilizes semi-solid behaviour and reduces macrosegregation, porosity and forming forces during the shaping process. Microstructural and mechanical characterization of 0.3, 0.5 and 1.0 wt% graphene nanoplatelet (GNP) reinforced A356 aluminium alloy composite fabricated by thixoforming was investigated. Stir casting was employed to fabricate feedstocks before they were thixoformed at 50% liquid. The microstructure was characterized and evaluated by field emission scanning electron microscopy with an energy dispersive X-ray detector and X-ray diffraction. Mechanical testing, such as microhardness and tensile testing, was also performed to estimate the mechanical properties of the composites. The incorporation of 0.3 wt.% GNPs in Al alloy increased by about 27% in ultimate tensile strength and 29% in hardness. The enhancement in tensile strength is primarily attributed to load transfer strengthening due to the uniform dispersion of these GNPs within the Al matrix, which promotes effective load transfer during tensile deformation, and GNPs’ wrinkled surface structure. Simultaneously, the addition of GNPs enhances the grain refinement effect of the Al alloy matrix, resulting in a grain size strengthening mechanism of the GNPs/Al composites. The results reveal that thixoformed composite microstructure consists of uniformly distributed GNPs, α-Al globules and fine fibrous Si particles. The composites’ grains were refined and equiaxed, and the mechanical properties were improved significantly. This study creates a new method for incorporating GNPs into Al alloy for high-performance composites.

## 1. Introduction

Aluminium alloy plays a crucial role in various fields such as the automotive and aerospace industries due to being an excellent lightweight structural and functional material [1,2]. However, its strength cannot meet the developing needs of the industry [3,4]. Therefore, aluminium matrix composites (AMC) are increasingly attractive because of their outstanding properties, such as low thermal expansion, high specific modulus and specific strength, and good wear resistance [3]. 

The addition of reinforcement such as silicon carbide (SiC) [5,6], boron carbide (B_4_C) [7] alumina oxide (Al_2_O_3_) [8] are common in AMC while graphene [9,10] and carbon nanotubes (CNTs) [11,12,13,14] which are made up of carbon-based nanomaterials, have attracted tremendous attention for AMC. Graphene nanoplatelets (GNPs) have become an excellent reinforcement because of their superior mechanical, thermal and electrical properties [15,16,17] as compared with CNTs. This material exhibits ultra-high tensile strength up to 130 GPa, an elastic modulus of 1 TPa, a low thermal expansion coefficient and high thermal conductivity [18,19]. In addition, graphene has a 2D sheet-like structure with a higher surface area and cheaper production cost than CNTs, thus making it an excellent alternative reinforcing material for composites. However, the non-wetting characteristics of the surface of GNPs have always resulted in severe issues with their dispersion in an aluminium matrix.

Various other processing methods have been developed to produce GNPs/Al composite, such as ultrasonic stirring, spark plasma sintering [20], hot extrusion process [21] and semi-solid metal processing (SSMP). Powder metallurgy has been favoured over other methods to achieve a uniform dispersion of GNPs in the aluminium because of its low processing temperatures, sufficient dispersion and low aggregation of the reinforcements [21,22]. However, it is not economical and has limitations in the fabrication of intricate parts. In this regard, stir casting processing is a cheaper and more suitable route for complex designs and bulk composite production [13]. Large specific surface area and attractive Van der Waals interactions between graphene layers may still make it ineffective for uniformly dispersing GNPs in the metal matrix. This process cannot overcome the agglomeration issue. Therefore, methods which combine several processing stages have been proposed in order to reduce the GNP agglomerations, such as SSMP. Thixoforming is a type of SSMP that is well known for producing dense microstructures, less porosity, short cycle times and large components with complex shapes, and it has been considered a potential route for processing AMCs [23,24]. These features make thixoforming a favourable choice for processing AMCs. A successful key to this technique is to get a billet with a non-dendritic microstructure, which can be easily obtained by reheating the composite at a semi-solid temperature within the range of 30–50% liquid fraction. Thus, liquid thixoforming has been proposed in this current work to allow a uniform distribution of reinforcing particles and a dense microstructure [18]. 

Several groups have investigated the optimum graphene content for Al matrix composite. Wang et al. investigated the graphene effect on the microstructure and thermal, electrical, mechanical and anticorrosive properties by Field Activated and Pressure Assisted Synthesis (FAPAS). They found that 0.5 wt% of GNP content was uniformly dispersed at grain boundaries in the Al matrix. As a result, the percentage increment of tensile strength, hardness and corrosion resistance are 30.6%, 44%, and 31%, respectively, as compared with pure Al. However, as the graphene content exceeds 0.5 wt.%, agglomeration occurs at grain boundaries in the Al matrix, leading to a decrease in each property [25]. Meanwhile, Li et al. [3] used a two-step processing method including cryomilling and a hot extrusion process to prepare the graphene/aluminium composite. They found that 0.5 wt% of graphene successfully increased strength and unsubdued ductility over pure aluminium. Furthermore, if the graphene nanoflakes were above 1.0 wt.%, the strengthening effect was significantly reduced because of the graphene agglomeration. Rashad et al. [26] investigated GNP-reinforced Al using a semi-powder method followed by hot extrusion. They found that 0.3 wt.% GNP was distributed homogeneously in Al and exhibited +11.1% and +14.7% higher UTS and yield strength, respectively, and had −40.6% lower failure strain. 

The influence of graphene nanoplatelets on mechanical properties and microstructure fabricated by the thixoforming process has not been extensively studied. Therefore, an experiment on a thixoformed GNPs/Al composite with variation in the GNP content is worth performing. First, the composite was reinforced with GNP content (at 0.3, 0.5 and 1.0 wt.%) via mechanical stir casting and then processed by thixoforming. GNPs were selected as reinforcement because of their ultra-high strength and multiply wrinkled graphene nanoplatelet structure resulting in increased strength and ductility as compared with monolithic aluminium [3]. The optimum GNP content as the function of the expected enhancement, the main strengthening mechanism of the GNP particles and the microstructure evolution were discussed. 

## 2. Materials and Methods

### 2.1. Materials

A commercially available A356 aluminium alloy (Al-0.7Si-0.3Mg) was selected as the matrix in this experimental study. It is a cast grade aluminium alloy supplied in the form of an ingot. The chemical composition of the A356 alloy obtained from optical emission spectroscopy (OES-Oxford) is tabulated in Table 1, which shows Silicon and Magnesium as its major alloying elements. GNPs were obtained from Sigma-Aldrich (St. Louis, Missouri, USA) with purity >95%, particle size of <2 um and average thickness of a few nm. Mg powder with purity >99% was used as a wetting agent. Different ratios of GNPs (0, 0.3, 0.5 and 1.0 wt.%) and 1 wt% Mg were used for the experiment. 

### 2.2. Composite Fabrication 

The mechanical stir casting route was employed to fabricate the GNPs/Al composite to produce non-dendritic feedstock for thixoforming. First, the GNP powder and 1 wt.% of Mg were weighed using an analytical balance (Mettler Toledo, AB54-SRS, Germany) before being mixed. Then, about 400 g of the Al-7Si alloy matrix was melted in the induction furnace at 700 °C. The temperature decreased to 650 °C before the reinforcement powder was injected via a hollow stainless steel rod. The rod acted as a plunger, pushing the reinforcement powder into the bottom of the crucible. The liquid mixture was then mechanically stirred at 500 rpm for 5 min using a three-blade radial impeller. Finally, the molten composite was poured into a 150 °C preheated mould of 25 mm and 120 mm in diameter and height, respectively, to cast the composite billets. The composite billets were made with the addition of 0.3 wt.%, 0.5 wt.% and 1.0 wt.% of GNPs.

Thixoforming was performed by placing the produced billet into the induction coil with an inner diameter and height of 100 mm and 200 mm, respectively, by using a T30–80 kHz 35 kW thixoforming machine. Each composite billet was machined to 25 mm and 100 mm in diameter and length, respectively, before being placed within the induction coil. Next, the billet was rapidly heated at 130 °C/min to prevent unfavourable grain growth. Once the billet reached a temperature of 580 °C, at 50% liquid fraction, it was maintained isothermally for 5 min to allow for spheroidization of the primary phase [27,28]. To monitor the temperature, a K-type thermocouple was inserted into a hole drilled at the top of the billet’s centre to a depth of 4 mm. The thermocouple was then removed before the billet was forged into the die using a laboratory press with a load of 20 kN and a speed of 85 mm/s. Moreover, the applied pressure was held for 1 min after the press.

### 2.3. Analysis of Microstructure 

**Figure 1:**
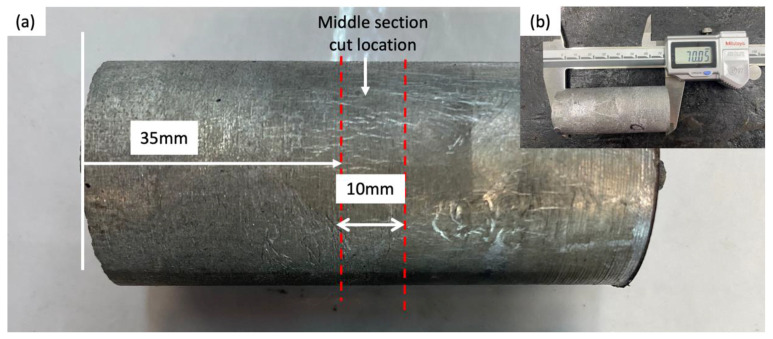
Whole sectional macrographs of thixoformed A356 aluminium matrix composite component reinforced by 0.3 wt.% GNPs (**a**) sample cut location; (**b**) whole length of billet composite (about 70 mm).

The samples used for each process were sectioned in a middle region of about 10 mm for metallographic examination, as shown in Figure 1. Microstructural analysis was performed by field emission scanning electron microscopy (FESEM- ZEISS Sigma 500, Germany) with an energy dispersive X-ray spectrometer operating at 20 kV. The standard metallographic procedure was conducted as follows for FESEM: surface grinding at different grit sizes (320, 600, 800 and 1200), polishing with diamond fluid (6, 3 and 1 μm) and etching with Keller’s solution agent for 10 s. Phase characterization results from thixoforming were conducted on the polished specimens by X-ray diffraction (XRD) D8 Advance X-ray diffractometer (Bruker AXS, D8 Advance, Karlsruhe, Germany) for which the system employed a Cu source and K-α as a radiation source. A characteristics study of the GNPs was undertaken by transmission electron microscopy (TEM) (Thermo Fisher Scientific, Talos L120 C, Waltham, MA, USA) at an accelerating voltage of 200 kV.

### 2.4. Mechanical Properties 

**Figure 2:**
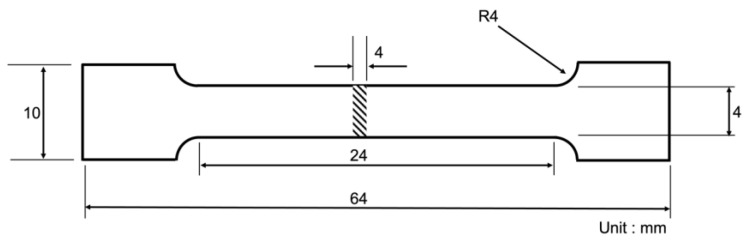
Schematic of a tensile test specimen.

The tensile test was carried out for each composite based on the ASTM E8M standard for the yield strength (YS) at 0.2% strain offset, ultimate tensile strength (UTS) and elongation to fracture. Tensile analysis was accomplished using a universal testing machine (Zwick, ProLine100, Lübeck, Germany) at a crosshead speed of 1.2 mm/min. The test was performed following the ASTM E8M standard. Tensile properties represent the average of three successive test results. Figure 2 displays the tensile test sample with a gauge length and diameter of 24 mm and 4 mm, respectively. The fractographic analysis was conducted on the fractured surfaces of GNPs/Al composites. It was examined by FESEM to understand the fracture mode during the tensile testing. The hardness of the composites was measured using a Vickers hardness tester (Zwick, ZHVµ, Lübeck, Germany) with an indenter load of 30 gf and a dwell time of 10 s.

### 2.5. Differential Scanning Calorimetry 

**Figure 3:**
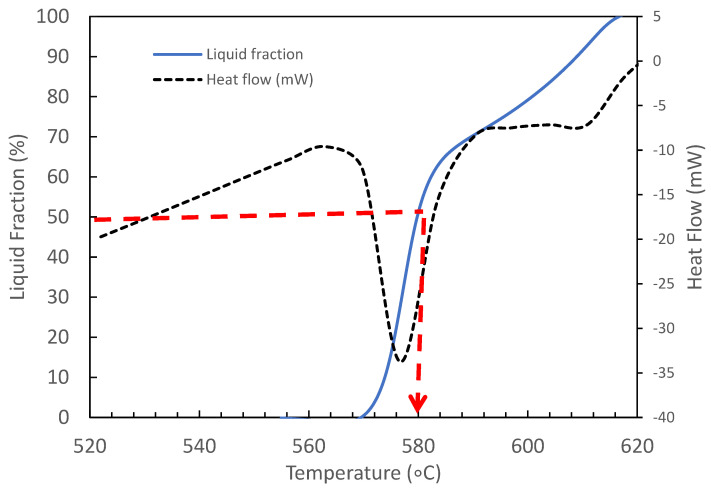
DSC and liquid fraction curve of 0.3 wt.% GNP/Al composite.

The heat flow and liquid fraction versus temperature of an A356 aluminium alloy/GNP composite was determined by the differential scanning calorimetry (DSC) technique which is shown in Figure 3. Differential scanning calorimetry analysis was performed on a Mettler Toledo TGA/DSC1 in an argon-controlled environment. A high-purity alumina pan was used as a reference material. The mass of the specimen used for DSC analysis was around 10 mg, and the sample was cut from the centre of the as-received alloys. The sample was then heated to 700 °C with a heat rate of 10 °C/min and purged with a neutral nitrogen gas atmosphere to prevent severe oxidation. The temperatures at the beginning and the completion of solidification are called the liquidus temperature T_liquidus_ and the solidus temperature T_solidus_, respectively. The solid line is the liquid fraction curve and the dashed line is the heat flow. A solidus temperature of 568 °C and liquidus temperature of 616 °C were achieved. The thixoforming involves reheating the billet from the solid to the semi-solid region. Thus, it shows that the semi-solid region of 30–50% liquid fraction is between 577–580 °C. Therefore, the thixoforming temperature would suggest that 580 °C was the maximum temperature before the liquid fraction of 50% was exceeded.

## 3. Results

### 3.1. Characterization of Raw Materials

**Figure 4:**
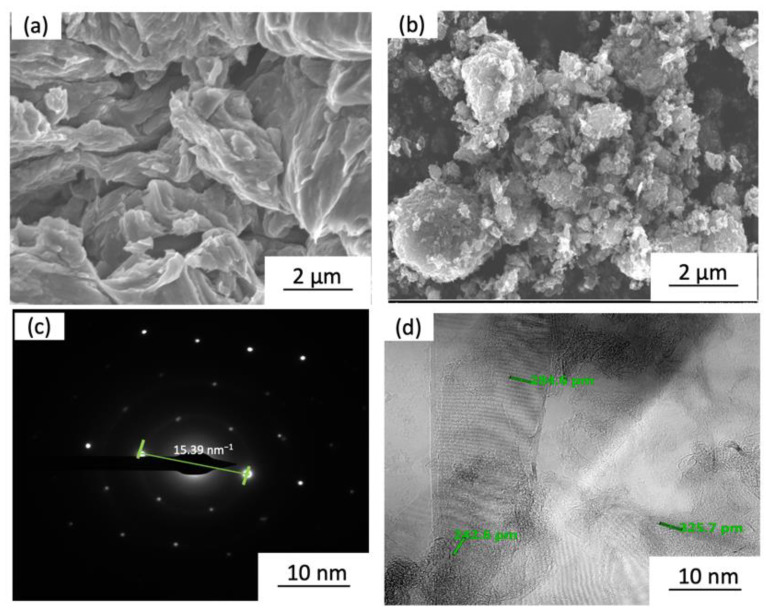
FE–SEM images of (**a**) Al alloy A356, (**b**) Graphene nanoplatelets (GNP_S_), and (**c**) SAED pattern of GNPs; (**d**) TEM images of as-received GNPs.

Figure 4 shows the images of the A356 alloy and GNP powders. Figure 4a depicts the morphology of the aluminium alloy and Figure 4b shows the plate-like structure of GNPs. The selected area electron diffraction (SAED) pattern is shown in Figure 4c which represents a single set of hexagon diffraction pattern characteristics. Meanwhile, the high-resolution TEM image of as-received GNPs in Figure 4d indicates the thickness and shows that the graphene’s surface is wrinkly, with several layers, and that the surface morphology is wavy folds. Microstructural investigation shows that each layer’s average thickness dimension of GNPs is 0.2–0.3 nm. 

### 3.2. XRD Analysis of Al-GNPs Composite 

**Figure 5:**
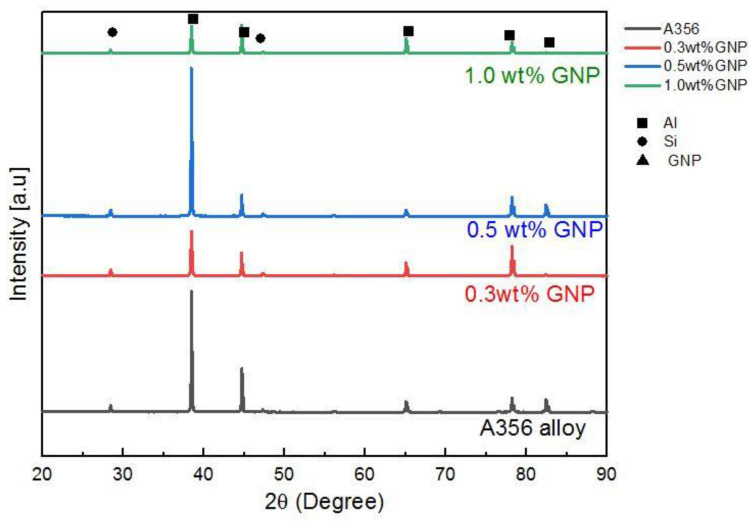
XRD pattern for GNPs/Al composites with variation in GNP content.

Figure 5 illustrates the XRD spectra of the Al monolithic alloy and Al-GNPs composites consisting of disparate weight fractions of GNPs. There are several peaks comprising peaks of Al and Si. The distinct peaks correspond for Al detected at several places such as at 38.47°, 44.72°, 65.10°, 78.23° and 82.44°. However, no peaks of GNPs are observed because of the low graphene content and the limited resolution of elements of X-ray diffraction. Research studies by Shao et al. [29] and Lou et al. [30] have also reported these findings on the absence of GNPs. In addition, there was no obvious peak of the aluminium carbide (Al_4_C_3_) phase found, thus proving that there was no chemical interaction between GNPs and Al alloy. Hence, this suggests that high-quality GNPs/Al composites were fabricated by the thixoforming process. The billet was reheated to a semi-solid temperature before the formation process, and thus the GNPs did not react with the aluminium on the grain boundaries.

### 3.3. Microstructural Analysis of Al-GNPs Composite

**Figure 6:**
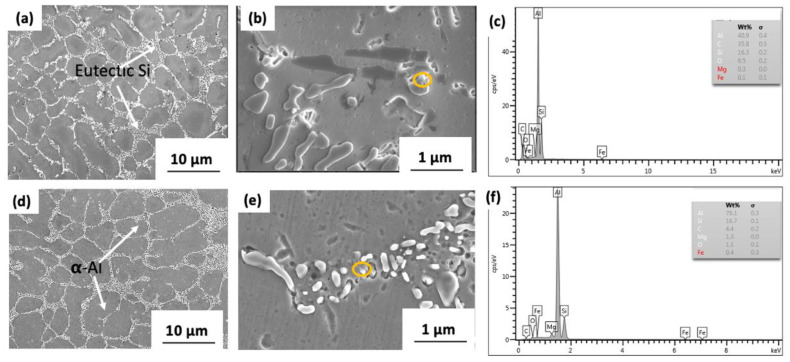
Backscattered images of thixoformed A356 alloy with the addition of (**a**) 0.3 wt.% GNPs and (**b**) high magnification images of 0.3 wt.%, (**c**) EDS spectrum for selected area (yellow circle), (**d**) 1.0 wt.% GNPs, (**e**) high magnification images of 1.0 wt.%, (**f**) EDS spectrum for selected area (yellow circle).

Figure 6 illustrates the backscattered diffraction images of thixoformed GNPs/Al composite with the addition of 0.3 and 1.0 wt% GNPs and the EDS spectrum. Thixoforming is an effective method for optimising the microstructure which has benefits in refining the grain and improving the material’s properties [24,31]. The microstructure of each composite was composed of eutectic silicon, α-Al and intermetallic compound. It is shown in Figure 6a,d that the grain of 0.3 wt.% GNPs is finer and more equiaxed than that of 1.0 wt.% GNPs. Hence, these findings suggest that low graphene content can promote grain refinement to obtain a high-strength GNPs/Al composite. The addition of graphene hindered the diffusion of Al atoms and refined the grain of the GNPs/Al composite, resulting in increased hardness and tensile strength (see Figure 9 and Figure 10).

Meanwhile, the microstructure α-Al phase in the thixoformed composite becomes more globular with increasing GNP contents. Furthermore, grain refinement improves the strength and plasticity as the grain size is in microns [15]. The backscattered electron microscopy images reveal the distribution of eutectic Si (light grey areas indicated with white arrows), α-Al matrix (a dark grey area) and EDS spectrum of the selected area in Figure 6c,f. 

**Figure 7:**
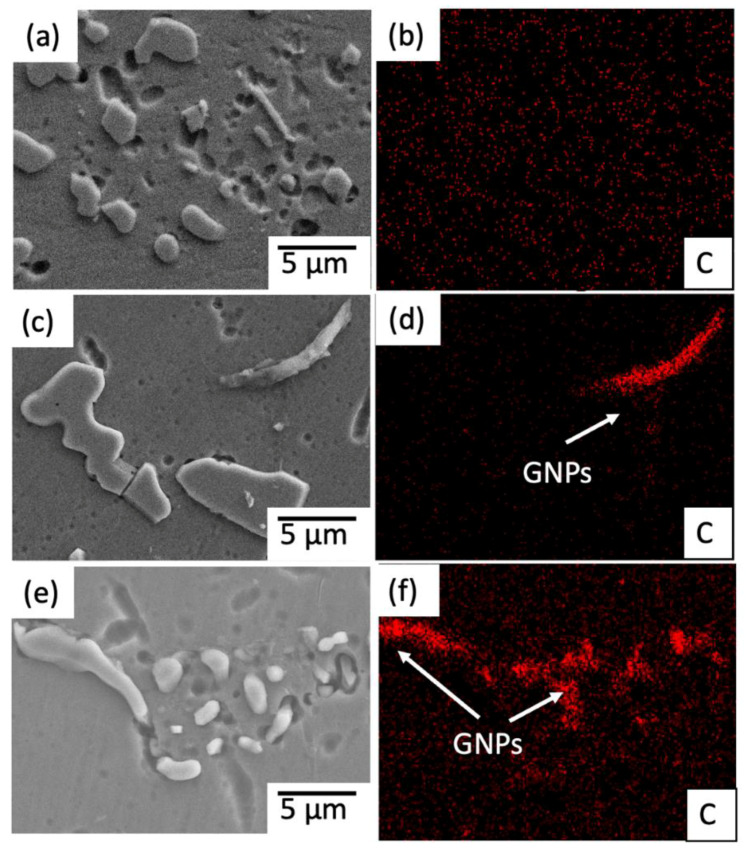
SEM images and their respective C element of thixoformed microstructure (**a**,**b**) 0.3 wt.% GNPs, (**c**,**d**) 0.5 wt.% GNPs, (**e**,**f**) 1.0 wt.% GNPs/Al composite with their respective C element.

Figure 7 exhibits the SEM images and element maps for each composite. The corresponding element of C referring to GNPs for each 0.3, 0.5 and 1.0 wt.% GNPs/Al composite is presented in Figure 7b,d,f, respectively. Homogenous distribution of GNP nanoparticles in the Al matrix was observed in the sample with 0.3 wt.% GNPs, as shown in Figure 7b. The great tensile strength attained by these composites further supported the strong bonding at the interface between the aluminium matrix and GNP. However, the GNPs adhere to the Al matrix surface because of the thixoforming process. It is worth mentioning that the illustrated elemental mapping C has proven the uniform and even distribution of the GNPs in the fabricated Al-GNPs composites. 

On the other hand, Figure 7d,f indicate a high accumulation of carbon (C) in the red strip-like phase as GNPs. Agglomeration of GNPs at several locations is observed for 0.5 wt.% and 1.0 wt.% of GNPs/Al composites as a result of a high percentage of GNPs, where the liquid metal has segregated the graphene particles and impeded them from mixing with the aluminium matrix [24,29].

### 3.4. Density and Hardness of Al-GNPs Composites

The theoretical, experimental and relative density values of the thixoformed composite are presented in Table 2. The theoretical densities of composites were calculated using the rule of mixtures, with the densities of aluminium and GNPs assumed to be 2.7 g/cm^3^ and 2.25 g/cm^3^, respectively. Meanwhile, the experimental density was measured by the Archimedes principle as shown in Equation (1).

(1) $$\rho_{c}=\frac{\rho_{w}\times W_{ac}}{W_{ac}-W_{wc}}$$

In this expression, $\rho_{c}$, $\rho_{w}$ denote the density of composite and the density of distilled water, and $W_{ac}$, $W_{wc}$ the weight of the composite in air and the weight of the composite in water. Adding graphene has increased the density of Al alloy owing to its low theoretical density. A similar trend in the density results was reported by Wang et al. on Al-Graphene composites [25]. 

**Figure 8:**
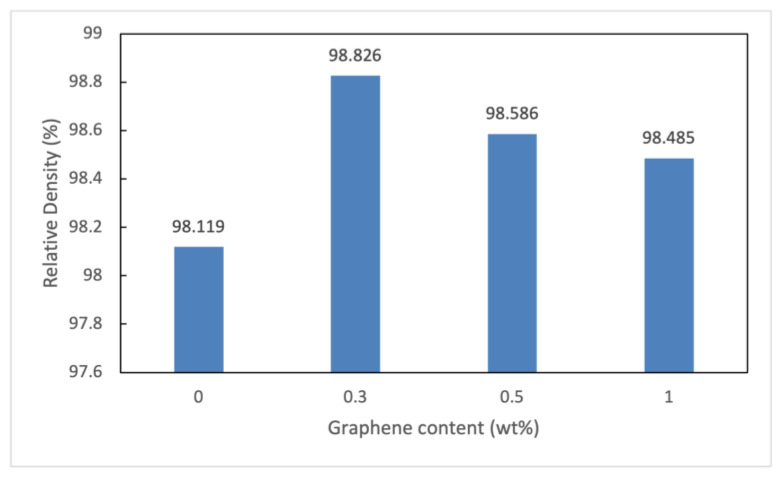
Relative density of thixoformed composite sample.

Figure 8 shows the relative density of composites which is essential for determining the performance of a material. The relative density ($\rho_{r}$) of the developed composites was determined using the measured density ($\rho_{m}$) and the theoretical density values ($\rho_{t}$) as indicated in Equation (2). The relative density of 0.3 wt.% GNPs/Al composite is the highest at 98.82%, suggesting that it has the lowest porosity and the densest as compared with others. The relative density decreases significantly with increases in graphene content. The presence of pores and agglomeration of graphene sheets at the grain boundaries of the Al matrix can cause a reduction in relative density [25]. The GNP cluster was found in composites containing 1 wt% GNPs, which were responsible for the existence of porosity and decrease in the relative density. The evidence of the presence of clusters may be witnessed in Figure 7d,f, which also show the cluster encapsulating the smaller aluminium particles. These clusters cause a decrease in the density of composites and the effects appear significantly at higher values of GNPs.

(2) $$\rho_{r}=\frac{\rho_{m}}{\rho_{t}}\times 100$$

**Figure 9:**
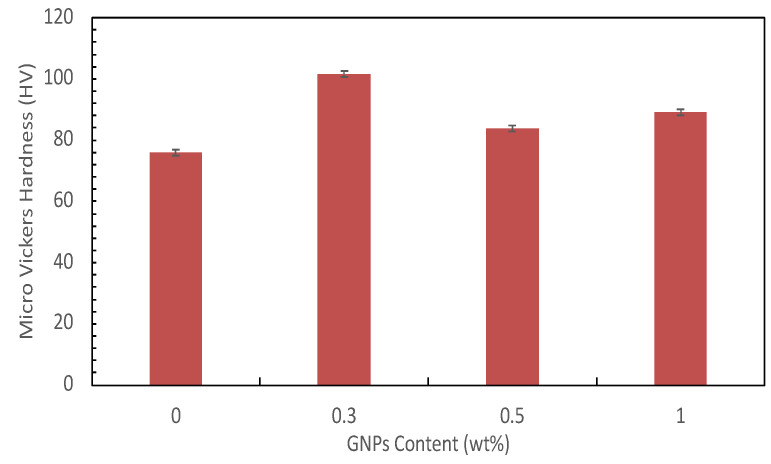
Comparison of the hardness of alloy and composite with different compositions.

Figure 9 shows the hardness of each composite at varying GNP contents. Overall, the composites’ hardness value is superior to that of the Al alloy. The addition of GNPs has a significant influence on the hardness increment. The hardness was enhanced by more than 29% when 0.3 wt.% GNPs were added, from 78 HV to 101 HV. However, as the GNP content increased, the hardness value tended to decrease. This trend is highly associated with changes in density and the microstructures. As discussed earlier, the 0.3 wt.% GNP/A356 composite with the highest relative density exhibited the highest hardness.

### 3.5. Mechanical Properties and Fracture Mechanism

**Figure 10:**
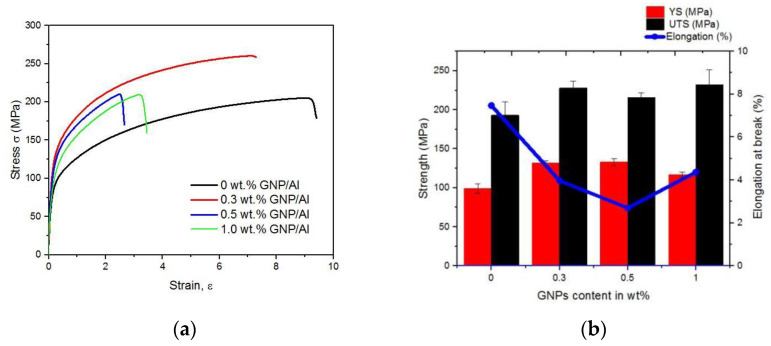
Tensile properties of Al alloy and GNPs/Al composites with different graphene contents. (**a**) Tensile stress–strain curve; (**b**) relationship between fracture elongation and YS, UTS.

The tensile stress–strain curve of Al and composites with variation in GNP content are shown in Figure 10a. Hence, the ultimate tensile strength (UTS), yield strength (YS) and fracture elongation are shown in Figure 10b to further analyse the trend changes in mechanical properties of GNPs/Al. The value of UTS, YS and elongation are summarised in Table 3. It is seen that the UTS and YS of nanocomposites compared with unreinforced alloy received remarkable enhancements. As compared with Al alloy, the ultimate tensile strength of 0.3, 0.5 and 1.0 wt% GNPs/Al composites increases by 27% (246.5 MPa), 11% (215.0 MPa) and 19% (231.6 MPa), respectively, and the yield strength increases by 43% (141.5 MPa), 34.6% (132.67 MPa), 19% (117.3 MPa), respectively. The addition of 0.3 wt.% of GNPs improves the ultimate tensile strength and yield strength by almost 50% of that measured for Al alloy.

On the other hand, the fracture elongation decreased by 30%, 47% and 41% for 0.3 wt.%, 0.5 wt.% and 1.0 wt.%, respectively. The 0.3 wt.% GNPs/Al composites maintain the favourable ductility of the A356 alloy as compared with the other composites. This suggests that adding 0.3 wt.% of GNPs is sufficient to preserve the ductility. Furthermore, it is noticed that the fracture elongation of the composites is linked inversely to their UTS with increasing graphene content. Typically, such extraordinary features are attributed to the synergistic interactions between evenly dispersed GNPs and ultrafine-grained Al matrices [25].

**Figure 11:**
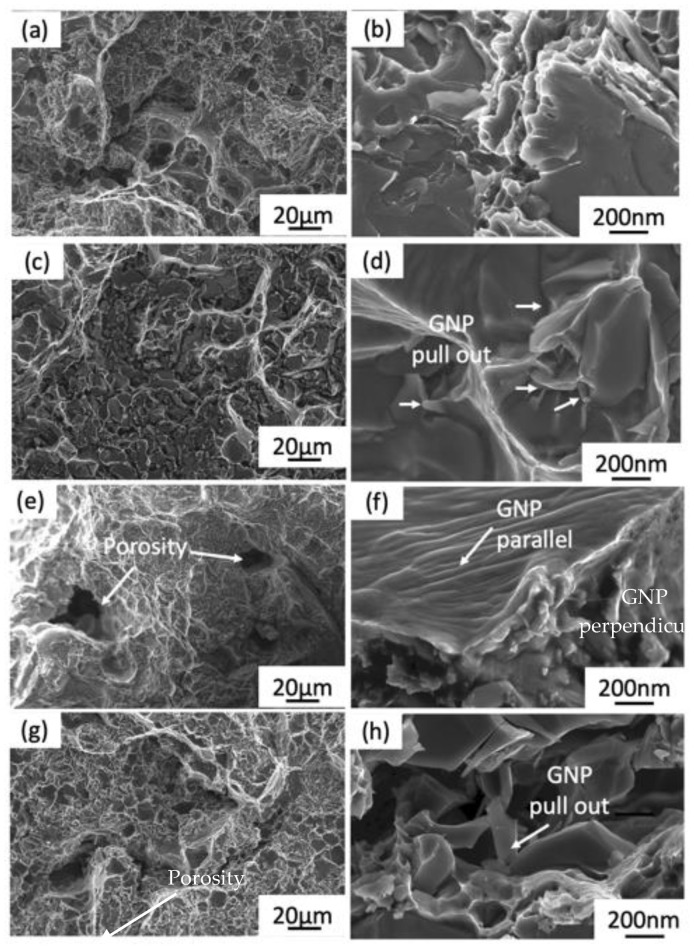
The FESEM fractured surface morphology and high magnification images of the GNPs/Al composites at various graphene contents: (**a**,**b**) 0 wt.% GNPs; (**c**,**d**) 0.3 wt.% GNPs; (**e**,**f**) 0.5 wt.% GNPs; (**g**,**h**) 1.0 wt.% GNPs.

The fracture surface morphology of Al alloy and GNPs/Al composites are shown in Figure 11. Figure 11a is the fracture profile of Al alloy and the fracture shown is a typical dimple formation type. Figure 11b–g shows the fracture morphology of 0.3, 0.5, and 1.0 wt.% GNPs and their high magnification, respectively. The dimples slightly decrease with the increases in GNP content and porosity, and are more evident in 0.5 wt.% and 1.0 wt.%. The change in fracture surface was accompanied by a decrease in elongation, as described in Table 2. The decrease in dimples and elongation as GNPs increased were also observed by Li et al. [32]. It is found that the fracture characteristics of 0.3 wt% composite changes the dimple size and depth with the bright ridges as shown in Figure 11c. This indicates the ductile failure characteristics consistent with high percentage of elongation obtained in 0.3 wt.% GNPs. GNPs are observed to be either parallel or perpendicular to the load direction, as shown in Figure 11f, further confirming the random distribution of GNPs in the matrix. 

Meanwhile, porosity was observed on the fracture of the 0.5 wt% and 1.0 wt% GNPs/Al composites in Figure 11e,g, thus indicating insufficient interface bonding between GNPs and Al composite and early failure of the composites. The crack propagation around the GNPs forms dimples of different sizes and shapes and some of them show tear ridges. In addition, it can be viewed on the higher magnification images, as shown in Figure 11d,h, that some GNPs were pulled out (marked by the white arrow in the figure), which serves in transferring the load. Further, this phenomenon helps to impede the propagation of cracks. Therefore, it is confirmed that effective bonding occurs between GNPs and the Al matrix. The analysis of fracture morphology establishes the properties of the composites.

### 3.6. Strengthening Mechanism

The addition of GNP nanoparticles has contributed to the enhancement of properties in composites because of several strengthening mechanisms. The strengthening mechanism of graphene reinforcement is assumed to be related to the unique structural characteristics of graphene, good bonding interfaces between graphene and aluminium and excellent mechanical properties.

Thermal mismatch strengthening, load transfer mechanisms, the Orowan strengthening mechanism and grain refinement can be attributed to the strengthening enhancement in mechanical properties [26]. The coefficient of thermal expansion of graphene nanoplatelets is 1 × 10^−6^ K^−1^, the same as graphite. On the other hand, aluminium has a coefficient of thermal expansion of 23.6 × 10^−6^ K^−1^. As a result, GNPs/Al composites have a significant coefficient of thermal expansion mismatch, resulting in prismatic dislocations punching at the interface, leading to composite matrix strengthening. The following formula [27] can be used to calculate the contribution of the thermal mismatch mechanism to tensile strength:

(3) $$\Delta \sigma_{thermal}=kG_{m}b\;\sqrt{12\frac{\Delta T\;\Delta C\;V_{g}}{bd_{g}}}\;$$

where *k* is a constant (0.5), $G_{m}$ is the shear modulus of the matrix (27.5 GPa), *b* is the Burgers vector of matrix Al (0.286 nm), ∆*C* is the thermal coefficient expansion (CTE) difference between matrix and reinforcement, and ΔT is the temperature difference between the thixoforming process (580 °C) and ambient temperature 25 °C. In addition, the *V_g_* is the volume fraction of GNPs and *d_g_* is the mean particle size of the GNPs. 

The Shear lag model [26] can explain the load transfer from the matrix to reinforcement. Load transfer from the matrix to reinforcement is primarily determined by interfacial bonding between the matrix and reinforcement caused by interfacial shear stress. Figure 7b shows that GNPs are uniformly embedded in the Al matrix, resulting in efficient load transfer from the matrix to reinforcement and increased composite strength. The following Equation (4) can be used to calculate the increase in yield strength of composites caused by load transfer.

(4) $$\Delta \sigma_{LT}=\frac{V_{gnp}\;\sigma_{m}}{2}\;$$

where $\sigma_{m}$ is the strength of matrix and $V_{gnp}$ is the volume fraction of GNPs. The Orowan strengthening mechanism also plays a significant role in strength enhancement. The nanoparticles in the composite can inhibit dislocation movement and enhance the materials’ strength. According to Zhang et al., the presence of highly dispersed nanosized reinforcement particles (smaller than 100 nm) in a metal matrix makes Orowan strengthening preferable in AMC due to the resistance of closely spaced hard particles to the dislocation passage. The following Equation (5) can be used to calculate the increase in yield strength of composites caused by Orowan strengthening.

(5) $$\Delta \sigma_{Orowan}=\frac{0.13\;G_{m}b}{d_{r}\left[{\left(\frac{1}{2V_{g}}\right)}^{1/3}-1\right]}\ln\frac{d_{r}}{2b}\;$$

where *G_m_* and *b* are the shear modulus and the Burgers vector of matrix Al. *V_g_* is the volume fraction of reinforcements in the composite. 

The grain refinement mechanism is another possible strengthening mechanism for AMCs reinforced by GNPs. The finer the grain, and the more grain boundaries, the greater the resistance to dislocation motion. Because of dislocation pile-up concentrated at the grain boundaries the stress concentration was calculated theoretically. The Hall–Petch Equation (6) describes the grain-boundary strengthening mechanism as shown below [28].

(6) $$\Delta \sigma_{GR}=\sigma_{0}+\frac{k_{y}}{\sqrt{d}}$$

where $\sigma_{0}$ is the friction stress, *d* is the average grain diameter and $k_{y}$ is the Hall–Petch slope. This effect is mainly related to the inhibition of the reinforcement on grain growth during the thixoforming process.

The enhancement in strength is caused by adequate bonding and dispersion of GNPs between the reinforcement and matrix. Apart from that, the thixoforming process contributes to a more refined and compact primary α-Al grain distribution, which reduces the crack initiation and porosity, which leads to the increment in tensile strength, as shown in Figure 7a. Based on the previously described Hall–Petch relationship, a refined grain increases the tensile strength since a globular grain is more equiaxed than a columnar grain, which would produce higher strength with reduced elongation [33]. The deformation or shear force caused by the thixoforming process separates the dendritic arm and multiplies the grain [31,34]. Billet reheating promotes the formation of globular grain structures. The globular grain structure and the refinement of the grain as the effects of the thixoforming process were proven to increase the mechanical properties of Al alloy [28,31,35]. 

Load transfer from aluminium to high-strength graphene is made possible by high interfacial strength. The Hall–Petch effect could also be responsible for this increment in composite strength. Chak et al. and Boostani et al. found similar results in the load transfer strengthening mechanism in their research findings [17,36]. In contrast to other composites, the agglomeration of GNPs caused the composite to fail earlier (1.0 wt.%). The refined grain and strengthening effect of GNPs are responsible for the improvement in the mechanical properties of composites reinforced with GNPs. During the deformation course of the composites, the reinforcement is strengthened by the Orowan strengthening effects, increased dislocation density, and load-bearing effects. This is because the GNPs in the current work are a few nm in size, and thus the Orowan strengthening mechanism works more effectively.

## 4. Conclusions

The effects of GNP on the microstructure, mechanical properties and strengthening mechanism of GNPs/A356 composite with a variation in the number of GNPs prepared by stir casting and thixoforming were successfully investigated. It is worth noting that the 0.3 wt.% GNPs/Al alloy nanocomposites have a significant advantage in ductility, strength and hardness. The thixoforming process is beneficial for a homogenous distribution of GNPs, resulting in a 47% and 23% increment in yield strength and ultimate tensile strength, respectively. The wrinkled structure of GNPs and effective bonding may be attributed to this behaviour. During plastic deformation, the wrinkled structure was straightened. When further increased in deformation, this promotes GNPs pulling out from the matrix. 

Even though some encouraging results have been obtained, unexplored influences of interfacial reaction and structural integrity in GNPs/Al composites still need to be investigated. Therefore, our future study will concentrate on enhancing the properties by precipitation-hardening heat treatment to increase the strength and improve the dispersion of GNPs in the metal matrix.

The formation of phase composites having an agglomeration effect with increasing concentration of GNPs is confirmed by the structural (XRD) and compositional analysis (EDX). The morphologies of eutectic Si in different processes are noticeably different. The thixoforming process promotes a smaller and compact eutectic structure. The hardness increases with up to 0.3 wt% GNPs and then decreases. Therefore, the GNP/A356 composite exhibits an improved tensile strength (from 193 MPa to 246 MPa) and yield strength (from 98 MPa to 141 MPa) and maintains good ductility simultaneously. 

## Figures and Tables

**Table 1:** A commercial A356 alloy matrix composition by weight fraction (wt.%).

Si	Mg	Fe	Cu	Mn	Ti	Zn	Al
7.27	0.32	0.185	0.006	0.004	0.119	0.009	Balance

**Table 2:** Theoretical density, experimental density and relative density of thixoformed Al-GNPs composites.

GNP Content (wt.%)	Theoretical Density (g/cm^3^)	Experimental Density (g/cm^3^)	Relative Density (%)
0 wt.%	2.700	2.649 ± 0.004	98.119
0.3 wt.%	2.698	2.666 ± 0.006	98.826
0.5 wt.%	2.697	2.659 ± 0.018	98.586
1.0 wt.%	2.695	2.695 ± 0.002	98.485

**Table 3:** Mechanical properties of GNPs/Al composites.

Samples	Ultimate Tensile Strength (MPa)	Yield Strength (MPa)	Elongation (%)
Al alloy	193.0 ± 17.0	98.5 ± 6.0	7.46 ± 3.18
0.3 wt% GNPs/Al	246.5 ± 19.0	141.5 ± 2.6	5.25 ± 2.62
0.5 wt% GNP/Al	215.0 ± 6.0	132.6 ± 4.6	3.96 ± 0.20
1.0 wt% GNP/Al	231.6 ± 9.3	117.3 ±3.2	4.36 ± 1.39

## Data Availability

The data presented in this study are available in this article.
